# A Delphi Method Analysis to Create an Emergency Medicine Educational Patient Satisfaction Survey

**DOI:** 10.5811/westjem.2015.10.28291

**Published:** 2015-12-11

**Authors:** Kory S. London, Bonita Singal, Jennifer Fowler, Rebecca Prepejchal, Stefanie Simmons, Douglas Finefrock

**Affiliations:** * Thomas Jefferson University, Philadelphia, Pennsylvania; † University of Michigan, Ann Arbor, Michigan; ‡ St. Joseph Mercy Hospital, Ann Arbor, Michigan; § Hackensack University Medical Center, Hackensack, New Jersey

## Abstract

**Introduction:**

Feedback on patient satisfaction (PS) as a means to monitor and improve performance in patient communication is lacking in residency training. A physician’s promotion, compensation and job satisfaction may be impacted by his individual PS scores, once he is in practice. Many communication and satisfaction surveys exist but none focus on the emergency department setting for educational purposes. The goal of this project was to create an emergency medicine-based educational PS survey with strong evidence for content validity.

**Methods:**

We used the Delphi Method (DM) to obtain expert opinion via an iterative process of surveying. Questions were mined from four PS surveys as well as from group suggestion. The DM analysis determined the structure, content and appropriate use of the tool. The group used four-point Likert-type scales and Lynn’s criteria for content validity to determine relevant questions from the stated goals.

**Results:**

Twelve recruited experts participated in a series of seven surveys to achieve consensus. A 10-question, single-page survey with an additional page of qualitative questions and demographic questions was selected. Thirty one questions were judged to be relevant from an original 48-question list. Of these, the final 10 questions were chosen. Response rates for individual survey items was 99.5%.

**Conclusion:**

The DM produced a consensus survey with content validity evidence. Future work will be needed to obtain evidence for response process, internal structure and construct validity.

## INTRODUCTION

The quantification of patient satisfaction (PS) data has become its own industry. Physicians’ pay, promotion and job satisfaction may be influenced by PS scores. Residents will be expected to practice independently in this environment, yet they are given little objective patient feedback on the care they provide. This limits the opportunities they have for improvement while in training.

The Council of Residency Directors for Emergency Medicine (CORD-EM) created a taskforce with the mission of creating a database of PS education and evaluation resources. This includes creation of a free, open-source survey instrument to provide resident feedback. Emphasis was placed on behavioral traits that affect patient satisfaction scores which are amenable to remediation. Importance was also placed on its validity.

The concept of psychometric validity is itself a controversial subject in survey development. There is no single reliable measure of validity; it is instead the sum of multiple facets that provide evidence of what has been deemed ‘construct validity.’ These include content validity: “whether [the survey] covers a representative sample of the behavior domain to be measured.”^1^ The goal of this project was to create and provide content validity evidence for an emergency medicine-based, educationally grounded PS survey.

## METHODS

We used a method of survey development created by the RAND Corporation, called the Delphi Method (DM) analysis. This process involves gathering experts and using iterative, anonymous surveying to determine consensus. It has been used by others to create PS surveys.^2^,^3^ The goal of the method is to achieve consensus through rounds of advocacy and opposition, hopefully minimizing the influence of strong but prejudiced or ill-informed opinions. This study was reviewed by the institutional review board and found to be exempt.

Given the differences in geographic practice patterns, experts were solicited from across the United States. Our goal was to recruit a diverse expert group of educators, residents and administrators with PS-oriented careers (see Appendix I). These include national emergency medicine leadership (the 2014–2016 American Academy of Emergency Medicine President and 2013–2015 CORD PS taskforce chair), emergency physician PS researchers and educators, residents with interest and experience in PS research and hospital administrators with PS expertise. These 12 hail from seven states (Colorado, Wisconsin, Michigan, Tennessee, Georgia, New York and New Jersey) and include three residency program directors and three assistant program directors. The average clinical experience of the attending physician experts was 8.6 years post residency training with a median of 9.5 years.

Potential survey items included in the analysis were chosen from four patient satisfaction tools (see Appendix II).^4^–^7^ Qualitative questions were taken from the author’s previously published work.^8^ Six additional questions were also suggested by the experts themselves given concerns that some essential aspects were not represented on the initial question list. Given the desire for readability, small grammatical changes were made so that all items followed the same syntax.

Left undefined by the DM analysis is the definition of expert consensus. The seminal works in this field are by Lynn and Lawshe.^9^–^10^ Both advocated for four-point scales, with low values denoting disagreement with the content, high values infer the opposite. Lynn recommended three or more experts with decreasing benefit from very large numbers. Lawshe created a table of critical values of agreement depending on the number of participants (up to 40 experts). For Lynn, content validity is defined as agreement by ≥80% of experts, Lawshe required lower rates for groups >8 members (for instance, a 12-member panel would require 56% agreement^10^). Given the more stringent requirements of Lynn’s criteria, they were chosen to define consensus and establish content validity for our survey.

The surveying itself was performed using the online, anonymous survey service Survey Monkey. Surveying was split into three series. The “initial series” surveys were focused on determining the tool’s structure and individual question content validity (relevance). The “second series” surveys chose which items from the “initial series” made it into the final product. Finally, a single survey was sent following the completion of the process to evaluate for expert approval with the final product.

The data was analyzed by the authors using Microsoft Excel and the built-in tools from the SurveyMonkey website.

## RESULTS

The experts chose a single-page, 10-item survey. Demographic questions about the patient’s age and gender were included. Additional questions about global satisfaction with the physician’s care as well as the satisfaction with the other facets of the patient’s visit were chosen for comparison. Both patients themselves as well as family members were allowed to participate. Given concern for consent, it was decided only patients and family members aged 18 or older would be eligible for participation. A second optional page, with qualitative questions and additional demographic data was recommended for inclusion. The tool was entitled BOOST: Behaviorally Oriented, Open Satisfaction Tool.

Forty-two items were chosen from the initial sources.^4^–^8^ From expert comments, six additional items were added. Of these 48, 31 were found relevant in the “initial series.” During the initial “second series” survey, three items tied for tenth place. Two sets of two similarly themed items were present in those 13. Therefore two redundant items were dropped and the three items that tied for tenth place were all included.

Seven surveys were required to complete the Delphi Method analysis. These included four “initial series” (which took place from 9/14-12/14), two “second series” (12/14-1/15) and the final affirmation survey (4/15). All experts participated in every survey, giving a 100% overall response rate. There was a 99.5% individual response rate for each survey item.

## DISCUSSION

With the increasing influence of the PS industry, educating the next generation of physicians on effective practice habits is integral to their success. Furthermore, high PS score have been shown to improve rates of patient compliance,^12^ a goal of all physicians. PS scores also inversely correlate with rates of litigation, another important aspect of a successful clinical career.^13^ Finally, PS techniques can provide comfort and minimize suffering of patients, a core tenet of medicine.^14^

Central to the idea of skill improvement is the ability to receive feedback. This survey’s content validity and focus on behavioral traits can provide actionable data and allow for credible improvement or remediation plans. Before this survey is ready for use we anticipate the need for two further steps: 1) Evaluation of survey readability and comprehensiveness from the patient perspective using focus groups; and 2) In situ investigation of BOOST in use with patient/resident dyads to determine inter-item agreement and correlation of multiple patient ratings of individual residents. This future work will further establish response process, internal structure and construct validity.

## LIMITATIONS

Two major limitations stand out. One was the relatively low number of experts, 12. With a larger number of experts for the DM, we may have elicited a different set of questions, or included additional survey items touching on different areas. There is some evidence, however, to indicate that a larger sample of experts may not lead to further response diversity once a threshold is reached and our threshold of validity (80%) was higher than Lawshe would require (56%) for a group of 12 experts.^9^.^10^ The second is that our list of items did not include questions on timeliness of care or pain management as mentioned in a comprehensive review.^11^ The former was left off secondary to concerns that timeliness is more of a systems issue than the responsibility of a resident. The latter was left off given concerns of how opioid utilization has fueled an epidemic of addiction that left some experts uncomfortable using pain control as a quality metric. Two authors also participated as experts (Finefrock and Simmons) but did not take part in data analysis and only helped create the research protocols and write the final manuscript.

## CONCLUSION

We developed a draft survey with content validity evidence using a DM analysis. It was based on initial questions with high content validity as many had been developed from literature review of patient-preferred behaviors or been validated in prior studies. Our group of experts spanned a large geographic and professional spectrum, increasing the generalizability of the study results. The questions are largely behavioral, creating practical data for educational purposes. Qualitative questions were provided on an optional basis. These can provide context and other data that quantitative analyses sometimes miss, though require greater patient effort and time utilization. Further work is needed to attain the high level of construct validity required for use in educational settings.

## Supplementary Information


