# Supplementary material for: A Delphi Method Analysis to Create an Emergency Medicine Educational Patient Satisfaction Survey
Source: West J Emerg Med. 2015 Dec 11;16(7):1106–8. doi: 10.5811/westjem.2015.10.28291 (PMC4703152; doi:10.5811/westjem.2015.10.28291)
Supplement: Supplementary file 1 [file wjem-16-1106-s001.docx]

Appendix 1

**Key:**

- Patient Satisfaction Questionnaire 3 – PSQ3
- Patient Experience of Care Survey - PEoC
- Communication Assessment Tool - CAT
- Patient Set - PS
- London Qualitative Study
- Questions suggested by the experts - QSE

**Final Questions with Sources:**

1. This doctor greeted me in a way that made me feel comfortable. (CAT)
2. This doctor introduced him/herself so that my guests and I knew who they were. (PS)
3. This doctor listened carefully to what I have to say. (PSQ-3)
4. This doctor respected my privacy. (QSE)
5. This doctor treated me with courtesy and respect. (adapted from PS)
6. This doctor explained things in a way that was easy to understand. (PEoC)
7. This doctor told me about the results of my medical tests. (PEoC)
8. This doctor involved me in decisions as much as I wanted. (CAT)

1. I would recommend this doctor to a friend or loved one. (QSE)
2. The care this doctor provided makes it more likely I would return to this hospital if needed. (QSE)

**Full List of Survey Items for the Delphi Method Analysis with Sources:**

1. This doctor allowed me to say everything that I think was important for this visit. ^PSQ-3^
2. This doctor asked me about my pain. ^PEoC^
3. This doctor greeted me in a way that made me feel comfortable. ^CAT^
4. This doctor introduced themselves so that I [and my guests] knew who they were. ^PS^
5. This doctor apologized if I felt my wait time was too long. ^PS^
6. This doctor sat down while talking to me. ^QSE^
7. This doctor needed to be more thorough in treating and examining me.^PSQ-3^
8. This doctor spent plenty of time with me. ^PSQ-3^
9. This doctor spent enough time with me. ^PEoC.^
10. This doctor spent the right amount of time with me.^CAT^
11. This doctor involved me in decisions as much as I wanted. ^CAT^
12. This doctor shook my hand or made physical contact with me before examining me. ^PS^
13. This doctor tried their best to control my pain. ^PS^
14. This doctor offered or did something for me that wasn’t related directly to my care (a blanket, food or water, dimmed the lights, made bed more comfortable, etc.). ^PS^
15. This doctor respected my privacy. ^QSE^
16. This doctor made me feel foolish. ^PSQ-3^
17. This doctor who treated me showed genuine interest in me as a person. ^PSQ-3^
18. This doctor should have given me more respect. ^PSQ-3^
19. This doctor showed me respect and treated me with dignity. ^PEoC^
20. This doctor treated me with respect. ^CAT^
21. This doctor ignored what I told them.  ^PSQ-3^
22. This doctor listened carefully to what I have to say.  ^PSQ-3^
23. This doctor used medical terms without explaining what they meant. ^PSQ-3^
24. This doctor talked in terms I could understand.  ^CAT^
25. This doctor did their best to keep me from worrying. ^PSQ-3^
26. This doctor encouraged me to ask questions. ^CAT^
27. This doctor let me speak without interruption. ^CAT+PS^
28. This doctor was good about explaining the reason for medical tests. ^PSQ-3^
29. This doctor explained things in a way that was easy to understand. ^PEoC^
30. This doctor told me what my medical problem was. ^PEoC^
31. This doctor told me about the results of my medical tests. ^PEoC^
32. This doctor told me how I could improve my medical condition and feel better. ^PEoC^
33. This doctor asked me if I had any questions about my care. ^PEoC^
34. This doctor checked to be sure I understood everything. ^CAT^
35. This doctor discussed next steps, including any follow up plans. ^CAT^
36. This doctor informed me about the amount of time my testing and care might take. ^PS^
37. This doctor came to update me about the status of my work up and care. ^PS^
38. This doctor explained why I was either being discharged or admitted to the hospital. ^QSE^
39. This doctor acted too businesslike and impersonal towards me. ^PSQ-3^
40. This doctor treated me in a very friendly and courteous manner. ^PSQ-3^
41. This doctor showed interest in my ideas about my health. ^CAT^
42. This doctor paid attention to me (looked at me, listened carefully). ^CAT^
43. This doctor smiled and made eye contact with me. ^PS^
44. This doctor showed care and concern. ^CAT^
45. This doctor understood my main health concerns. ^CAT^
46. I am confident I will not have to come back to the emergency department for this problem. ^QSE^
47. I would recommend this doctor to a friend or loved one. ^QSE^
48. The care this doctor provided makes it more likely I would return to this hospital if needed. ^QSE^

**Qualitative Questions**

1. Please provide any additional comments that you would like us to know. ^PEoC^
2. What did your doctor do well? ^Qualitative Abstract^
3. What could your doctor improve upon? ^Qualitative Abstract^
4. What are the most important parts of being a great doctor? ^QSE^

**Demographics:**

1. Gender (Male/Female/Other/Do not wish to answer).
2. What is your Age (21 and under, 22-34, 35-44, 45-54, 55-64, 65 and over).
3. The problem I came to the emergency department for is entirely new for me.
4. How many times have you visited an emergency department in the last year?
5. My chief complaint was:
